# Year-round high physical activity levels in agropastoralists of Bolivian Andes: Results from repeated measurements of DLW method in peak and slack seasons of agricultural activities

**DOI:** 10.1002/ajhb.20864

**Published:** 2009-01-06

**Authors:** Hiroshi Kashiwazaki, Kazuhiro Uenishi, Toshio Kobayashi, Jose Orias Rivera, William A Coward, Antony Wright

**Affiliations:** 1 Department of Human Sciences, School of Health Sciences, University of Occupational and Environmental HealthKitakyushu, Fukuoka, Japan; 2 Laboratory of Physiological Nutrition, Kagawa Nutrition UniversitySaitama, Japan; 3 Graduate School of Health Science, Hiroshima UniversityHiroshima, Japan; 4 Hospital Virgen de CopacabanaLa Paz, Bolivia; 5 MRC Human Nutrition Research, Elsie Widdowson LaboratoryCambridge, UK

## Abstract

By the repeated use of the doubly labeled water method (DLW), this study aimed to investigate (1) the extent of changes in energy expenditure and physical activity level (PAL) in response to increased agricultural work demands, and (2) whether the seasonal work demands induce the changes in the fairly equitable division of work and similarity of energy needs between men and women observed in our previous study (Phase 1 study; Kashiwazaki et al., 1995: Am J Clin Nutr 62: 901–910). In a rural small agropastoral community of the Bolivian Andes, we made the follow-up study (Phase 2, 14 adults; a time of high agricultural activity) of the Phase 1 study (12 adults; a time of low agricultural activity). In the Phase 2 study, both men and women showed very high PAL (mean±SD), but there was no significant difference by sex (men; 2.18 ± 0.23 (age; 64 ± 11 years, *n* = 7), women; 2.26 ± 0.25 (63 ± 10 years, *n* = 7)). The increase of PAL by 11% (*P* = 0.023) in the Phase 2 was equally occurred in both men and women. The factorial approach underestimated PAL significantly by ≈15% (*P* < 0.05). High PAL throughout the year ranging on average 2.0 and 2.2 was attributable to everyday tasks for subsistence and domestic works undertaking over 9–11 h (men spent 2.7 h on agricultural work and 4.7 h on animal herding, whereas women spent 7.3 h almost exclusively on animal herding). The seasonal increase in PAL was statistically significant, but it was smaller than those anticipated from published reports. A flexible division of labor played an important role in the equitable energetic increase in both men and women. Am. J. Hum. Biol., 2009. © 2009 Wiley-Liss, Inc.

Since the doubly labeled water (DLW) method has been established as an important tool for the measurement of total energy expenditure (TEE) in man, there have been many reports detailing TEE from a wide range of population groups under free-living conditions. As reviewed by Black et al. (1996), the method has been applied in diverse circumstances ranging from premature infants to the extreme elderly, and from bed-bound patients to athletes performing at the limits of human endurance. However, these studies have been made predominantly in affluent groups, typically urban populations of industrialized societies.

In contrast, very few DLW studies have been performed in rural populations of developing countries. Currently reports are limited to those of Gambian farmers (women: Heini et al., 1991; Singh et al., 1989; men: Heini et al., 1996), Andean male and female agropastoralists in Bolivia (Kashiwazaki et al., 1995), male farmers in rural south India (Borgonha et al., 2000), Bangladeshi lactating women working for tea estates (Rosetta et al., 2005), and lactating women of 3-6 month postpartum from a Mexican subsistence community (Butte et al., 1997). Except for the latter study, a high physical activity level (PAL) ranging from 1.90 to 2.4 is found in these populations.

In general, these studies are cross-sectional and performed during a particular time of the year. Several earlier reports (Adams, 1995; Bleiberg et al., 1980; Brun et al., 1979, 1981; Ferro-Luzzi et al., 1990; Lawrence et al., 1987; Panter-Brick, 1993, 1996; Schultink et al., 1993) deduce that rural populations (particularly in non-industrialized societies) are exposed to seasonal energy imbalance, evidenced by body weight changes due to naturally occurring seasonal variations in food availability and to the changes in work demands for agricultural activities during the year. Although the reported PAL values obtained from DLW on nonindustrialized rural societies are more reliable than those derived from other methods, they do not provide a comprehensive picture by themselves. When seasonal changes in physical activity have to be considered, the estimate of energy requirements becomes even more difficult. Unfortunately there is little information available on extent of change in TEE and PAL between the peak and slack seasons of agricultural activity caused by varying obligatory tasks for subsistence.

The purpose of this study was to address this issue by comparing energy expenditure and PAL at two contrasting time and work intensity periods of the year in a rural agropastoral population. In 1990, we obtained data from an Andean Bolivian community during January and February, the preharvest season when labor demands for agriculture are at a minimum (Phase 1: Kashiwazaki et al., 1995). This work reports a return visit to the same community during the season of more intensive agricultural activity (August-September), when field preparation and potato planting increases the total number of daily tasks being undertaken, which is achieved in part by a redistribution of the work load between family members (Phase 2).

The repeated use of the DLW method in this rural community of the less developed world has allowed us to investigate firstly, whether energy expenditure intensifies markedly in response to increased agricultural activity, and secondly, whether the seasonal work demands induce the changes in the fairly equitable division of work and similarity of energy needs between men and women observed in the Phase 1 study. A timed-record of activities collected for 3—4 days during the DLW study were used as complementary information to examine these questions.

## SUBJECTS AND METHODS

The return visit to the same community as the previous study was made in August 1997 (Phase 2), a season contrasting to that of the previous study, as it is when the ground is leveled for potato planting. This season requires greater labor demands than the preharvest season of our previous Phase 1 study (Kashiwazaki et al., 1995). We anticipate a change in several aspects of labor demands between the two periods. The additional activities of field preparation is likely to promote a change or redistribution of work schedule for each of the family members, and an understanding of the impact of this on energy expenditure is of great interest in the realm of nutritional adaptation as well as clinical nutrition in rural populations of nonin-dustrialized societies.

### Subjects

This study was made in Vilacollo (Kashiwazaki et al., 1995), an estancia consisting nine households (eleven households in the year of 1990—1991) at high altitude (about 4,000 m above sea level) near the Bolivian border with Chile. The subjects are Aymara-speaking people living in a small community with outlying pastures and small fields for potato growing. The climate in this region is most severe from June through August, the temperature at night falling to between 0 and − 10°C, with daytime temperatures of 10°C and humidity less than 30%. In summer, December through February, nighttime temperatures still fall to between 0 and 3°C, with daytime temperatures reaching between 12 and 20°C.

Sixteen adult subjects (age greater than 18) and four children and adolescents were recruited to the 1997 study, representing eight of the possible nine households. Due to out-migration of two households and some young family members, five of the adult subjects were overlapped with the previous study (Kashiwazaki et al., 1995), and other adults were the subjects newly participated in this Phase 2 study. All procedures of this study were reviewed and approved by the Ethical Committee at the University of Occupational and Environmental Health, Kitakyushu (former affiliation of HK).

### Procedures of the DLW study

The procedures of the study were the same as those of our previous study (Kashiwazaki et al., 1995). Before the doubly labeled water was administered, subjects underwent a general health check, anthropometric measurements and were asked to provide a predose urine sample for baseline isotopic measurements. The body weight of subjects in minimal clothing was measured with a digital balance after subjects voided their bladders to the nearest 0.05 kg. Typical clothing was also weighed for correction to nude weight. Measured subject's weight was used to estimate the isotope dosing requirements. At the end of the study, no significant change in their body weight was detected.

Each subject received a mixed oral dose of ^2^H2^18^O containing 0.062 g ^2^H_2_O/kg and 0.149 g H_2_^18^O/kg. The subject was then given a cup of water or a light refreshment drink to ensure that the entire dose was consumed. Subjects were instructed to avoid large fluid and food intakes for the next 4 h, and the first postdose urine sample was collected after a 6-h equilibration period. For 14 days thereafter, urine samples were collected daily in early or mid morning after subjects had voided overnight urine. To avoid evaporation and isotopic exchange with atmospheric air, urine sample was immediately transferred in duplicate to a small air-tight vial (10 ml) closed by a screw cap, tightly sealed with silicon tape, and kept in a dark and cool room.

After the entire field research was completed, all urine samples were sent to Cambridge (UK) for stable isotope analysis.

### Resting metabolic rate

The postabsorptive resting metabolic rate was determined in duplicate and on two successive days in the early morning, according to standard procedures for measurement of basal metabolic rate (BMR). For simplicity and comparative purpose, we expediently defined and used these data as BMR in this article. The day before the BMR measurement, each subject assigned to the DLW study was asked to accept a visit by one investigator to his or her house in the early morning, at about 06:00, usually before the subject woke up. While subjects were remaining in their own bed at complete rest and after an overnight fast, a facemask was attached to them to collect expired air. The procedure of measurement was the same as that described previously (Kashiwazaki et al., 1986, 1990). No control was made for room temperature, which ranged from 4 to 8°C (outdoor temperature ranged from −5 to 2°C). During the measurement, subjects were laid on their bed and covered with sufficient clothing and blankets to provide a comfortable temperature of 25—28°C over most of their body. Only the subject's face was exposed to room temperature, which may have affected the measurement of BMR, producing a slightly higher value than that measured in controlled laboratory conditions (Kashiwazaki et al., 1986, 1990). These conditions were the same as those with the Phase 1 study (Kashiwazaki et al., 1995), providing the comparable BMR data. After 5 min of stabilization, expired air was collected with a Douglas bag, twice, for 5 min. Tests for leaks were made at each measurement. The volume of expired air was measured with the certified dry gas meter (Shinagawa Seisakusho, Tokyo), and the sample of expired air was analyzed for O_2_ and CO_2_ concentrations in duplicate with a Roken-shiki portable gas analyzer (Shimada, Tokyo; a modified Hal-dane gas analysis apparatus that can be used without a supply of electric power). BMR was calculated by using Weir's equation (Weir, 1949). The accuracy and precision of the apparatus were carefully checked in Tokyo before its application in the field. By simultaneous measurements of expired air with a paramagnetic oxygen analyzer and infrared carbon dioxide analyzer (Expired Gas Monitor, model 1H21; San-Ei Sokki, Tokyo), accuracy and precision was proved to be acceptable; differences in the measurements between the two apparatuses were negligible: the mean difference and 95% confidence limits of agreement for nine measurements of standard air were 0.08 ± 0.14% in oxygen and −0.12 ± 0.22% in CO_2_. Precision as CV was 0.4% for oxygen and 1.8% for CO_2_ on repeated measurements of expired gas. The measured BMR was highly correlated (*r* = 0.89, *P* < 0.001) with the BMR estimated from body weight by using the equations of the FAO/WHO/UNU (1985), and the mean differences on average of about 1% between the measured BMR and the estimated BMR was not statistically significant by paired *t*-test. In the Phase 1 study, measured BMR was 5.6% lower than estimated BMR (Kashiwazaki et al., 1995). This means that the calculated PAL (TEE/BMR) by using the measured BMR would include a potential underestimation by 4%–5% in the subjects of Phase 2 study.

### Measurement of isotopes and calculation of carbon dioxide production

^2^H and ^18^O composition of the urine samples, and samples of dilute doses were measured using a Sira 10 dual inlet mass spectrometer (Micromass, Wythenshawe, UK). For ^2^H, a 0.4 ml of urine was aliquoted into a disposable 3.5 ml glass vial containing a reusable platinum catalyst (Finnigan MAT, Bremen, Germany) and fitted with a rubber septum. Isotopic equilibration with hydrogen gas at 2 bars and 25°C and was complete after 3 h. The hydrogen was then admitted to the mass spectrometer for isotopic determination. Measurements were made against a sample of H_2_ gas similarly equilibrated with water of natural abundance and were corrected for interference of H^3+^. Laboratory standards calibrated with values of −51.45‰ and 763.24% relative to standard mean ocean water (SMOW)/standard light antarctic precipitation (SLAP) (147.75 and 274.64 ppm) were run as unknowns and the true enrichment of the analyzed samples calculated from these. Precision of the measurements was 1.58‰ (0.24 ppm). After use, the catalyst rods were washed with deionized natural abundance water and stored in air at 40°C for at least a day before reuse.

For ^18^O enrichments, 3 ml aliquots of urine were equilibrated with 13 ml CO_2_ at 400 mbars and 25 ± 0.1°C for 6 h on a shaker bench (Isoprep system Micromass, Wythenshawe, UK). After admission to the mass spectrometer, the CO_2_ was measured relative to a cylinder of gas traceable to international standards, and the isotopic composition expressed relative to SMOW. The precision of these measurements was 0.15‰ (0.3 ppm).

Dilution spaces for ^2^H (Nd) and ^18^O (No) and disappearance rate constants for ^2^H (Kd) and ^18^O (Ko) were calculated by the multipoint method (Coward, 1988). From these, calculation of CO_2_ production was made as previously (Kashiwazaki et al., 1995).

The respiratory quotient (RQ) required for the calculation of energy expenditure from CO_2_ production was assumed to be equivalent to the food quotient (FQ), which was taken as 0.93 as the mean estimate from our previous study (Kashiwazaki et al., 1995). Averaged 24 h total energy expenditure (TEE) was calculated from the mean daily CO_2_ production by using Weir's formula (Weir, 1949): kJ=LCO_2_ = 4.63 + 16.49=RQ

Propagation of error analysis was performed on each measurement to obtain an estimate of individual errors, which is the product of the standard errors of the flux rates (Ko and Kd), and the pool sizes (No and Nd) resulting in a standard error for the CO_2_ production uncorrected for fractionation (Cole et al., 1990). The errors computed by this procedure, contain analytical error and physiological variation from day-to-day changes in flux rates of O and ^2^H.

Total body water (TBW) determined by DLW was calculated from ^8^O and ^2^H dilution spaces combined. Fat-free mass (FFM) was estimated by dividing TBW by 0.732. Body fat was calculated by subtracting FFM from body weight.

### Timed-record of activities

Time-allocation study was conducted contemporaneously with the DLW study. A subset of six households composing of 15 subjects (12 adult subjects) was selected (four subjects from two households were not observed because of the remoteness of their houses from the majority of dwellings). Two well-trained assistants (both of them native speakers of Aymara, and one native to the area) observed the assigned subjects over 3–4 days. One observer was able to record usually two—three subjects of one household. The activities of each individual were recorded, and the times when changes in major activity occurred were noted. Records were kept from 6:30 am to 7:00–8:30 pm (about 12–14 h per day). Where there were missing periods or more detail required, the observations were augmented by ad hoc interviewing of the subject.

Recorded activities were classified into the following main categories and subcategories, I: subsistence (IA: daily herding and animal care, IB: farming work), II: household chores (IIA: cooking and food processing, IIB: washing and sweeping), III: eating and discretionary activities (IIIA: eating, IIIB: rest, chatting, and reading), IV: social relation (mainly time spent for attending the meeting with neighboring communities), and V: overnight sleep (when bed-time and wake-up time were missing, we assumed the rest of time was spent in overnight sleep). For each of the activity subcategories, the time weighted energy cost (EC_tw_) was calculated by using the energy costs (PAR: physical activity ratio), adopted from the published list of FAO (2004).

### Statistical analysis

All data are expressed as mean 6 standard deviation (SD), unless otherwise noted. Statistical analyses were performed by using SPSS 11J statistical software for Windows (SPSS Japan, Tokyo, Japan). Correlation analysis was used to assess the association between energy expenditure and other variables. Differences between measurements were analyzed by unpaired Student's *t*-test (pooled- or separate-variance estimates after homogeneity of variance test). To test the effect of factors (sex, phase; year, and interactions), least-squares means were also computed and adjusted for weight as a covariate by using the general linear models (GLM) procedure in group comparisons of the measurements on adult subjects. When the probabilities were <0.05, the statistical tests were regarded as significant.

## RESULTS

Table 1 shows individual data on physical characteristics, DLW variables, total energy expenditure (TEE), BMR, and PAL. Data on children and adolescents are also presented for reference. The following analyses are limited to those for adults. Although only 7 years elapsed between the two studies, the mean age of the adult subjects (male; 64 ± 11 years, female; 63 ± 10 years) resulted in the increase by about 25 years. This is partly due to the aging of overlapped subjects with the previous study, but mostly due to new subjects selected from the remained elderly people resulted from out-migration of the younger members. Medical checks made by one of the authors (JOR) before giving stable isotope found no severe health problems in any of the subjects, however, many of them complained of poor vision, and shoulder- or head-ache usually associated with fatigue.

**TABLE 1:** Individual data for doubly labeled water variables, energy expenditure, and post absorptive resting metabolic rate^a^

Subject	Age (year)	Height (cm)	Weight (kg)	Fat^b^ (%)	Ko (24 h^−1^)	Kd (24 h^−1^)	No (mole)	Nd (mole)	TEE (kJ/24 h^−1^)	Estimated error (%)	BMR (kJ/24 h^−1^)	PAL
Male												
M1	17	160.9	51.1	14.3	0.1379	0.1021	1792.1	1852.8	13058	16.1	6376	2.05
M2^c^	34	167.4	56.0	13.9	0.1558	0.1210	1964.7	2049.2	13736	7.1	6707	2.05
M3^d^	52	161.7	53.5	18.8	0.1353	0.1055	1770.7	1836.4	10544	5.9	5448	1.94
M4^d^	53	165.1	68.5	27.5	0.1431	0.1061	2026.6	2111.6	15351	4.5	6657	2.31
M5	55	160.3	52.0	9.8	0.1524	0.1164	1927.8	1979.0	13966	5.8	6443	2.17
M6	64	156.3	58.7	17.7	0.1346	0.1009	1972.4	2051.2	13556	5.0	5674	2.39
M7^d^	72	158.4	46.6	13.9	0.1339	0.1026	1634.8	1703.9	10305	5.8	5259	1.96
M8	75	164.0	57.3	21.1	0.1015	0.0736	1847.4	1916.7	10611	3.8	5372	1.98
M9	80	157.1	53.5	20.1	0.1512	0.1127	1718.5	1805.8	13569	5.2	5402	2.51
Female												
F1^e^	6	114.7	18.9	17.7	0.1923	0.1521	641.4	655.5	5114	7.3	3916	1.31
F2	14	152.1	46.3	33.8	0.1767	0.1310	1242.3	1308.6	11678	4.7	5435	2.15
F3	16	155.8	41.8	18.8	0.1481	0.1109	1367.6	1462.0	10519	6.1	5720	1.84
F4^c^	25	160.4	79.1	43.0	0.1265	0.0980	1835.4	1920.3	10539	5.2	7037	1.50
F5^d^	52	156.9	44.3	25.2	0.1355	0.1004	1358.9	1401.5	9711	4.3	4678	2.08
F6	52	159.0	52.5	27.2	0.1782	0.1358	1550.7	1632.7	13397	6.1	5092	2.63
F7	58	147.3	54.4	36.9	0.1571	0.1171	1402.2	1455.0	11431	4.2	4879	2.34
F8	65	155.3	44.1	26.2	0.1489	0.1108	1333.4	1378.2	10347	4.5	4305	2.40
F9	67	145.8	40.2	26.9	0.1408	0.1001	1200.5	1248.5	10134	4.8	4293	2.36
F10^d^	71	161.1	45.8	18.2	0.1389	0.1060	1524.1	1594.7	10163	5.5	5330	1.91
F11	79	147.6	39.3	24.4	0.1615	0.1196	1210.5	1261.0	10372	4.8	4996	2.08

a aKo and Kd, elimination rates of ^18^O and ^2^H, respectively; No and Nd, time-0 dilution spaces of ^18^O and ^2^H, respectively; FFM, fat-free mass; TEE, total energy expenditure; BMR, overnight fasting resting metabolic rate; PAL, Physical activity level = TEE/BMR. FFM calculated from ^2^H and ^18^O dilution spaces, derived by back-extrapolation of the isotope disappearance curves to zero time, ie, total body water = (Nd/1.04+ No/1.01)/2 and was divided by 0.732.

b (weight–FFM) × 100/weight.

c F4 and M2 were husband and wife not engaging in the agropastoral activities, without the domestic animals. M2 was working as a teacher at the elementary school of this area. The major physical activity of F4 was household chores. Their physical activity pattern differed from most of the other adults in this rural estancia. Eight months before, F4 was hospitalized fora surgical operation and treatment of injury suffered from an unfortunate accident of stone hitting on the head.

d Subjects participated in the previous study.

e After a few days of giving DLW, F1 had repeated attacks of diarrhea and was inactive during the DLW period. This was reflected in the relatively high water turnover rate as seen in Kd and Ko.

**Fig. 1:**
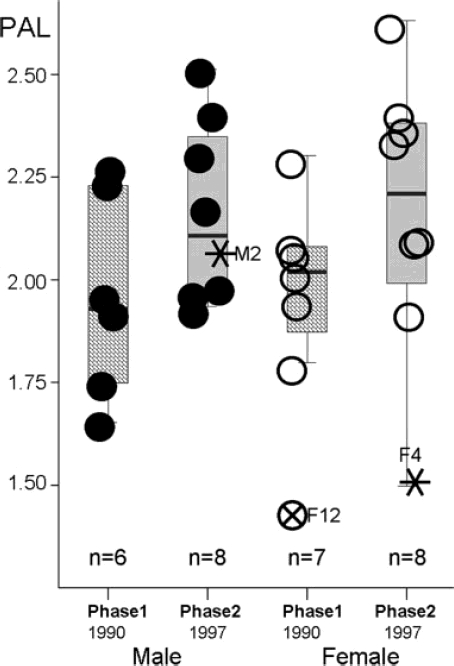
Physical activity levels (PAL) of adult subjects in Phase 1 (1990) and Phase 2 (1997). PAL: Total energy expenditure (TEE)/Basal metabolic rate (BMR). For details on subjects F12 (Phase 1), M2 (Phase 2), and F4 (Phase 2), see text.

An overview of the individual data for the adult subjects is shown in Figure 1; in which physical activity level (PAL: TEE/BMR) values are plotted in comparison with those from the Phase 1 study. In neither season was there a sex-difference in the PAL of adult subjects. In both male and female subjects, medians (indicated by gray bars in the box of the figure) shifted toward higher PAL in the season of Phase 2 (1997). As shown, two female subjects had PAL ≤ 1.5 extremely lower than other subjects. Subject F12 in Phase 1 (1990) was pregnant, and F4 in Phase 2 (1997) had been recently hospitalized and undergone surgery. She belonged to a household, which did not hold herds of animals, and her major physical activity was household chores. Her husband (M2) was working as a teacher at the elementary school of this area, and their lifestyle and physical activity pattern differed from most of the other adults in this rural estancia. Similar low PALs were found among Yakut men and women transitioning away from a subsistence herding lifestyle (Snodgrass et al., 2006). In the following analyses, subjects of M2 and F4 in Phase 2 (1997), and F12 in Phase 1 (1990) were excluded.

Body mass index (BMI), estimated FFM, and fat% suggest that the subjects have a normal body composition (Table 2). They appeared to have no tendency to an increased body fat with age as has been often observed in many societies of the developed countries (no significant correlations were observed between age and body composition; data not shown). Despite the advanced and older age of the subjects, when compared with the Phase 1 study, no statistical differences were detected in BMI, FFM, or fat% between Phase 1 and Phase 2.

**TABLE 2:** Physical characteristics of the adult subjects in 1990 and 1997

	Phase 1 (1990)				Phase 2 (1997)				Significance of effect^a^		
	Male *n*=6		Female *n*=6		Male *n*=7		Female *n*=7				
	Mean	SD	Mean	SD	Mean	SD	Mean	SD	Sex	Phase	Sex* Phase
Age (year)	41.5	14.8	38.2	18.7	64.4	11.4	63.4	10.0	ns	*P* = 0.000	ns
Height (cm)	161.9	5.7	153.9	5.3	160.4	3.4	153.3	6.3	*P* = 0.001	ns	ns
Weight (kg)	54.8	2.8	48.6	5.4	55.7	6.9	45.8	5.7	*P* = 0.001	ns	ns
BMI	21.0	1.6	20.5	1.9	21.6	2.3	19.5	2.7	ns	ns	ns
FFM (kg)	46.3	2.4	35.4	3.6	45.2	3.4	33.5	3.4	*P* = 0.000	ns	ns
Fat (%)	15.5	2.1	27.0	5.3	18.4	5.6	26.4	5.5	*P* = 0.000	ns	ns
BMR (kJ/24 h)	5669	501	4795	340	5751	563	4796	393	*P* = 0.000	ns	ns
BMR^b^ (kJ/24 h)	5067	200	5246	177	5254	175	5423	197	ns	ns	ns

BMI, body mass index expressed as weight/(height (m))^2^; FFM, fat free mass derived from dilution spaces of ^18^O and ^2^H. (for details, see Text and Table 1); Fat (%) = (Weight–FFM) × 100 / Weight.

a Statistical significance of the factor effects tested by GLM procedure.

b BMR adjusted for FFM as covariate and the data are adjusted mean ± SE.

ns, not significant; *P*, statistically significant with the probability of error.

Energy expenditure and physical activity indicators of the adult subjects were examined to test for the differences by both gender and phases (Table 3). Two procedures were applied. One was by comparing the least-squares means of TEE and activity energy expenditure (AEE, derived as TEE–BMR) with body weight as the covariate, as most activities are presumed to be weight bearing. The second was comparing TEE and AEE normalized by weight. Neither method indicated any gender differences, only differences between the two phases, where the extent of increase in the labor intensive season was almost identical for male and female adults, e.g., 11% increase in PAL and about 25% in AEE adjusted for weight.

**TABLE 3:** Comparison of energy expenditure and physical activity indicators of adult subjects by sex and year

	Phase 1 (1990)				Phase 2 (1997)				Significance of effect^a^		
TEE or Physical activity indicators	Male *n* = 6		Female *n* = 6		Male *n* = 7		Female *n* = 7		Sex	Phase	Sex* Phase
	Mean^b^	SE	Mean^b^	SE	Mean^b^	SE	Mean^b^	SE			
Adjusted Mean of TEE or AEE^b^											
TEE (MJ/24 h)	10.46	0.54	10.22	0.53	11.75	0.52	11.76	0.54	ns	P = 0.01	ns
AEE (MJ/24 h)	4.97	0.50	5.30	0.49	6.22	0.48	6.70	0.50	ns	P = 0.009	ns
	Mean^c^	SD	Mean^c^	SD	Mean^c^	SD	Mean^c^	SD			
Relative term (BMR or body weight)											
PAL (TEE/BMR)	1.96	0.25	2.04	0.17	2.18	0.23	2.26	0.25	ns	P = 0.023	ns
TEE/kg weight (kJ/24 h)	202	27	202	16	226	29	237	21	ns	P = 0.005	ns
AEE/kg weight (kJ/24 h)	99	26	102	14	122	24	131	19	ns	P = 0.005	ns

TEE, Total energy expenditure determined by Doubly labeled water method; AEE, Activity related energy expenditure derived as TEE-BMR.

a Statistical significance of the differences tested by GLM procedure;

b Mean and standard error (SE) adjusted for body weight as covariate. Adjusted mean was estimated with 51.2 kg body weight.

c No covariate was applied.

ns, not significant; *P*, statistically significant with the probability of error

Timed-activity data on 12 adults (six males and six females) allowed us to examine the physical activity patterns in detail, expressed as time spent and estimated time-weighted energy cost (EC_tw_) (Table 4). Both male and female subjects spent most of their time undertaking subsistence activities (male; 437 ± 70 min, female; 444 ± 92 min), which was about 30% (male; 30% vs. female; 31%) of time during 24 h. Of a total time spent on subsistence activity, men spent 2.7 h/24 h on agricultural work and 4.7 h/24 h on animal herding, whereas women spent 7.3 h/24 h almost exclusively on animal herding. In five major categories, although time spent in subsistence and sleep showed no gender differences, three activity categories (household chores, eating and other discretionary activities, and activity for social relations) had gender differences in allocated time. Gender differences in activity patterns appeared more obvious in subcategories. Women spent most of their time in daily herding and animal care, and rarely in other farming work. Compared with men, women spent more than twice the time undertaking household chores, less than half the time for light leisure time activities (chatting, rest, and reading), and little time in activities involving in the community meeting. When compared on the basis of EC_tw_, the results with respect to gender difference were the same as those times allocated. More than 50% (male; 56% vs. female; 52%) of average daily energy expenditure was spent on subsistence activities. For other everyday activities combined (activities II, III, and IV), EC_tw_ were 20% of TEE in male and 24% in female (sex difference was not detected). Sum of EC_tw_ did not differ between males and females. When EC_tw_ was transformed into estimated PAL_est_ (PAL_est_ = EC_tw_/24 h) derived from the timed-activity record and compared with the PAL derived from DLW, the values of PAL_est_ were significantly lower by «15% in both males (1.92 ± 0.12 vs. 2.21 ± 0.23, *n* = 6, *P* = 0.03 by paired *t*-test) and females (1.90 ± 0.09 vs. 2.24 ± 0.27, *n* = 6, *P* = 0.02 by paired *t*-test).

**TABLE 4:** Time spent (min/24 h) and estimated energy cost (PAR × h) for activities (Data from Phase 2)

	Time spent (min)										Time Weighted Energy Cost (EC_tw_) = Sum of PAR × time (hr)								
Activities Classified	Male				Female						Male				Female				
	Mean	SD	Mean	SD	Mean	SD	Mean	SD	Sex difference	Energy Cost (PAR^c^)	Mean	SD	Mean	SD	Mean	SD	Mean	SD	Sex difference
Five major categories																			
Sub-categories of the major categories																			
I: Subsistence	437.4	70			444.4	92			ns		25.7	4.9			23.8	4.8			ns
IA Daily herding and Animal Care			277	71			437	102	a	3.2			14.8	3.8			23.3	5.5	a
IB Farming work			160	100			8	18	a^d^	4.1			10.9	6.9			0.5	1.3	b^a^
II: Household chores	87	58			213.3	101			a		3.2	2.1			7.7	3.7			b
IIA food processing, and Cooking meals			43	34			146	59	a	2.1			1.5	1.2			5.1	2.1	a
IIB Sweeping, washing, repairing			44	27			68	51	ns	2.3			1.7	1.1			2.6	1.9	ns
III: Eating and discretionary activities	194.4	39			129.2	41			a		4.7	0.9			3.1	1.0			b
IIIA Easting meals, taking tea			80	22			75	13	ns	1.5			2.0	0.6			1.9	0.3	ns
IIIB Rest, chatting, reading			115	27			54	35	a	1.4			2.7	0.6			1.3	0.8	a
IV: Social Relation (Community meeting)	59	47			0	0			b^d^	1.4	1.4	1.1			0.0	0.0			b^a^
V: Overnight Sleep	662	74			653	85			ns	1.0	11.0	1.2			10.9	1.4			ns
Total	1440				1440						46.0	2.9			45.5	2.3			ns

a, b: by *t*-test for the differences, a; *P* < 0.01, *b* < 0.05; ns; not significant.

cPAR, Physical activity ratio (energy expenditure of the activity/BMR), adopted from Tables 5-1 FAO 2004.

d Denotes *t*-test by separate-variance estimate, after the F value used to test homogeneity of variance and its probability.

## DISCUSSION

High PAL of rural Andean Aymara has two notable features. Firstly, there was no decreasing trend in PAL with aging and senior adults over 60 years had PAL > 2. Secondly, the increase of work demands for agricultural activities resulted in an 11% increase in PAL of both men and women compared with the Phase 1 study (Kashiwazaki et al., 1995). This increase in PAL, or the extent of seasonal fluctuation, was smaller than those anticipated from published reports on agricultural communities of less developed world (Bleiberg et al., 1980; Brun et al., 1981; Heini et al., 1996; Lawrence and Whitehead, 1988; Singh et al., 1989). Particularly, important issues from this study are the high level of energy expenditure throughout the year, and the contrast between rural and urban lifestyle relevant to; (1) PAL with aging, (2) seasonality of physical activity and obligatory work demands, (3) out-migration of younger generation to cities, and (4) the importance of qualitative information on activity patterns, which the DLW study alone cannot provide (Durnin, 1996; Irwin et al., 2001).

There are a limited number of reports on TEE using DLW for healthy senior adults over 60 years old. For example, the DLW studies of 574 measurements compiled and summarized by Black et al. (1996) from the subjects of industrialized urban settings of affluent societies suggested a range of PAL between 1.2 and 2.5 for sustainable lifestyles, in which only six reports were for senior adults. Four reports targeted for healthy senior subjects living independently; had no participant aged over 80 years, and the PAL values ranged from 1.4 to 1.8. Higher PAL values were reported in subjects with a high level of leisure activity compared with other subjects of similar age, but a PAL of greater than 2.0 was rare even in the very active subjects. Physical activity patterns also differ from that of senior age groups in the urban industrialized world. In studies of European elderly subjects, most of time was spent laying and seated (70–80%), about 20% undertaking standing activities of light to moderate intensity, and less than 20% of time spent walking or performing recreational activities (Morio et al., 1997; Visser et al., 1995). Generally, the distribution of the PAL for the elderly is lower when compared with those younger than 65 years of age. The high levels of PAL in the rural agropastoral Aymara were mainly attributable to the every day tasks and subsistence activities necessary to maintain their domestic works and animal care etc, whereas high levels of PAL in senior citizens of the Western European society is limited to those who maintain a high level of leisure time activity.

### Year round high PAL and underlying factors

Seasonal fluctuations of work demands and food availability have been major issues when related to health and nutrition in rural communities of the less developed world. The variations in body weight, energy balance, and energy expenditure can result in long term weight cycling or the risk of shortage in energy intake, and this has been reported as having an adverse effect on health (Adams, 1995; Bleiberg et al., 1980; Brun et al., 1979, 1981; Ferro-Luzzi et al., 1990; Singh et al., 1989). These studies are in groups of subjects who exclusively rely on agriculture. In subjects of this study, no apparent nutrition-related health problems were observed. As indicated in Table 2, there was no significant difference in body weight, BMI, and fat% between the subjects of two phases. High PAL values similar to our subjects have been reported only from DLW studies in Gambian women (mean PAL 5 1.97; Singh et al., 1989) or Gambian males (mean PAL 5 2.4; Heini et al., 1996). These reports are based on research during the peak agricultural season. No report exists illustrating the extent of variation in PAL of rural subjects evaluated by DLW during the course of the year. In this respect, energy expenditure data measured on the Andean agropastoral Aymara subjects are unique in their high PAL throughout the year ranging on average between 2.0 and 2.2. With respect to the constancy of high PAL not only in middle-aged but also in the elderly men and women, the work demands for their subsistence activities and out-migration of younger generation to cities could have been the interrelated compounding factors.

### Relative importance of animal herding and agriculture for their subsistence

Animal care requires one or two of the family members to lead them from the corral to the appropriate grassplots at the extensive foothills of local mountains or pampas. At least one of the family members should watch and move the herds to other grassplots during the daytime, and then lead them back to the corral before sunset. Herding activity involves time-consuming tasks to watch grazing animals, and it is unwise to curtail time for this activity for two reasons. By watching animals and leading them to the appropriate grassplots, the herders prevent their animals from (1) overgrazing the patchy grassplots and (2) underfeeding and malnutrition of animals. Alpacas, llamas, and sheep are their primary wealth and source for their subsistence, providing them wools for textiles, exchanging outside foods, cash income, and dung fertilizer as well as supplying them food as meat. A sheep is generally slaughtered every 1—2 month for family consumption, or one llama/alpaca is consumed every 3—6 months. Shearing for wool from one animal happens every 2 years. A caravan of llamas is sometimes used to transport goods to an open-air market about 150—200 km away from this region. All family members know the necessity of providing animals with some level of constant care throughout the year, which is crucial for their survival under the harsh environmental conditions. Seasonal fluctuation in herding activity is less obvious than that of agricultural activity. A similar observation has been reported from the study in an agropastoral community in the foothills of Himalayas by Panter-Brick (1996), who concluded that seasonality as evaluated by time consumed for activity was highly significant for agriculture, forest work, and travel, but it was not detected for animal husbandry.

In contrast to animal herding, the relative importance of agriculture for their subsistence is much less than other rural community. Small plots, where soil and climatic conditions permit, are cultivated in expectation of a good harvest. The preparation of fields for planting potatoes and other feasible crops in this area is one of the few tasks that truly require the strength of adult males. However, heavy reliance on agriculture is perilous to people of this area because of the risk of damage to plants by unexpected frost and drought during the growing season. The harvest from the small plots of potato field is variable and generally not sufficient enough to support the annual food consumption in a household. It would cover only about 30–60% of needs. Kim et al. (1991) reported, based on a seven-day food consumption survey in September 1988 in this area, that locally produced foodstuffs such as potato, meat, and eggs provided 37% of total energy intake and that the foodstuffs produced outside the community were of critical importance for their food and nutrition. They relied heavily on animal herding for sources of wool and meat to bring cash income or would exchange them for potatoes, cereals, and other foods in the local market. No apparent changes in their lifestyle and the importance of outside foods were observed since 1988 or 1990.

### Out-migration of the younger generation

Out-migration of the younger generation of family members to attractive big cities, such as La Paz and El Alto, is another important factor leading both senior adult men and women to very high PAL. In a period of 10 years, since the household census survey of 1988, the population size of this community has decreased to 34% of 1988's total population. This decrease was exclusively due to the out-migration of the younger generation between 10 and 40 years at the time in 1988. The decrease of household members was substantial from 4.8/household to 2.9/household. Their subsistence and household activities, which had previously been shared among three—four household members, has become solely the tasks of the remaining household members; typically a husband and wife aged 50–80. If the additional work demands were only short-term and urgent, the logical solution would have been to recruit manpower from outside the household. However, all households, being virtually in the same situation with a shortage of manpower, have resulted in the only solution of existing members spending more time on subsistence and household chores. Thus, out-migration of the younger generation combined has been the compounding factors that underlie the senior adults having high physical activity levels. This may not be an aspect unique to this area, but also may be occurring in other rural communities in developing societies. The long-term consequences of out-migration and community aging in rural areas of developing societies, deserves much more attention from social and ecological perspectives of health, physical activity, and nutritional status.

### Importance of timed-activity records and limitations in estimating TEE

In many rural third-world communities, women contribute substantial time and energy to subsistence farming, even though they are childbearing (Panter-Brick, 1989). A study based on the observational timed-activity records, from rural areas of the Ivory Coast, reported that women consistently work more hours than men and spend less time on leisure activities (Levine et al., 2001). Identifying the use of time during 24-h, similar gender differences in time spent on work were observed in our Aymara subjects. Women spent 2 h more doing household chores and about 1 h less time on light leisure activities than men spent. The total time spent working on subsistence activities and household tasks was longer among women than men by 2.2 h (women; 657 min, men; 524 min). Observed differences in time allocated by men and women are one of the cross-sectional scenes during the year. As time needed for agricultural activities increases, men have to curtail their time spent on animal care and herding or other activities. Both husband and wife equally adjust and share their time to meet the extra work demands and because activities such as animal herding and care are crucial for their short-term survival, these tasks are unlikely to be curtailed to compensate. A flexible division of labor between husband and wife enabled the husband to engage in agricultural activities and to curtail his time for animal care. This results in an increased workload for both men and women; while men engaged in agricultural activities, women spent longer time in animal care and herding, hence compensating the time curtailed by the men. This brought almost equal increase of PAL (11%) and AEE (25%) derived from DLW data both in men and women. A similar pattern was observed in EC_tw_ estimated from the timed-activity records, in which the activities undertaking over many hours showed no significant energetic differences by sex. In rural areas of no electricity like this community, the total time available for subsistence working is governed by daylight hours, usually about 11 h in this season (sunrise at about 7:00 am and sunset at about 18:30). The time spent on subsistence activities and household tasks was 11 h in women and 8.7 h in men, suggesting very little margin of time available for additional work, particularly in women. The observed PAL during this period appears to represent their peak for the year.

Timed-activity records provide information not only on life-style and activity pattern, which DLW study alone cannot, but it is also used as the essential data to estimate energy expenditure in the factorial method. Several calculations, using the factorial approach, reported there was a systematic underestimation of PAL, which was ≈15%, both in men and women when compared with the DLW method. Similar or much greater levels of underestimation was also reported in studies validated by the DLW method (Haggarty et al., 1994; Roberts et al., 1991) and studies with heart rate monitoring (Leonard et al., 1995, 1997; Spurr et al., 1996). However, other recent DLW studies have reported that the factorial method provides a close estimate of TEE in groups of free-living adolescents (Bratteby et al., 1997) and elderly subjects (Morio et al., 1997), or in some cases, an overestimation of about 8% in males of 27—65 years (Conway et al., 2002; Irwin et al., 2001). Leonard et al. (1997) have pointed out, by reviewing studies on the factorial approach validated by DLW and HR methods, that underestimation with the factorial approach is more substantial at moderate to high activity levels than at very low activity levels. These and our report indicate that the factorial approach is prone to underestimate TEE. The reasons for systematic error remain to be examined further, but it must be emphasized that the observed underestimation in the factorial approach does not diminish the overall importance of timed-activity data.
